# Efficacy of intraoperatively prepared cell-based constructs for bone regeneration

**DOI:** 10.1186/s13287-018-1026-7

**Published:** 2018-10-25

**Authors:** Yang Zhang, Eline C Grosfeld, Winston A Camargo, Hongbo Tang, Angela M P Magri, Jeroen J J P van den Beucken

**Affiliations:** 1 Department of Biomaterials, PO Box 9101, 6500HB Radboudumc, Nijmegen, the Netherlands; 2 Department of Plastic Surgery, Tongji Hospital, Huazhong University of Science and Technology, Wuhan, 430074 China; 3 Department of Biosciences, Federal University of São Paulo, Santos, São Paulo Brazil

**Keywords:** Easy access cells, Intraoperative preparation, Stromal vascular fraction, Adipose-derived cells, Macrophages, Bone regeneration

## Abstract

**Background:**

Conventional cell-based bone regeneration suffers from the major disadvantage of limited cell supply, time-consuming in vitro expansion cultures, and limited patient-friendliness related to cell isolation and multiple visits to the clinic. Here, we utilized an alternative concept using “easy access cells” that can be obtained in an intraoperative manner to prepare cell-based constructs.

**Methods:**

We used stromal vascular fraction (SVF) from human adipose tissue and human monocytes for intraoperative preparation of bone constructs. Conventional constructs grafted with expanded human adipose tissue mesenchymal stem cells (ADMSCs) derived from the same donor were set as positive controls. Additionally, we combined both cell types either or not with monocytes. The cellular interaction of human SVF and ADMSCs with human monocytes was evaluated in vitro. The feasibility and bone-regenerative capacity of intraoperative constructs were determined histologically and histomorphometrically in a rat femoral condyle bone defect model.

**Results:**

SVF displayed equal in vitro osteogenic differentiation compared to donor-matched expanded ADMSCs, which for both was significantly enhanced upon co-culture with monocytes. Moreover, SVF and ADMSCs displayed different immunoregulatory effects on monocytes/macrophages. Upon implantation in rat femoral bone defects, SVF constructs demonstrated superior bone formation compared to ADMSC constructs and cell-free controls; no effects of monocyte addition were observed.

**Conclusion:**

In conclusion, we here demonstrate the feasibility of intraoperative SVF construct preparation and superior bone-regenerative capacity thereof compared to donor-matched ADMSC constructs. The superiority of SVF constructs was found to be linked to the distinct differences between immunoregulatory effects of SVF and ADMSCs.

**Electronic supplementary material:**

The online version of this article (10.1186/s13287-018-1026-7) contains supplementary material, which is available to authorized users.

## Background

Bone tissue engineering concepts have been proposed and conducted over the last decades to meet the increasing need for bone-regenerative therapies in clinics. Their development aims to overcome the limitations of allografts and autografts, which are restricted by limited supply, immunological rejection, and disease transmission issues [1, 2]. In bone tissue engineering-based constructs, mesenchymal stromal cells (MSCs) [3] have been mostly used as an autologous cell source to boost the bone regeneration process. Generally, these procedures involved bone marrow isolation, MSC expansion in vitro, MSC seeding on a scaffold material and priming osteogenic differentiation in vitro, and finally construct implantation into the bone defect of the patient [4]. This conventional approach has several drawbacks regarding tissue harvest via bone marrow aspiration and time-consuming expansion procedure due to low yield of MSCs in the bone marrow [5]. An alternative in the form of liposuction has become available after the first reports on the existence of high yield of MSCs within adipose tissue with osteogenic differentiation capacities [6, 7].

Although recent work has clearly demonstrated the feasibility and efficacy of using human adipose tissue MSCs (ADMSCs) to generate bone constructs in pre-clinical animal studies [8, 9], clinical application still is limited due to the disadvantages of the preparation process. To generate ADMSCs, the harvested heterogeneous cell population requires purification and expansion in vitro. In addition, inconvenience from two surgical procedures, for respectively cell harvest and construct implantation, is an obstacle. In order to attain simplicity, practicality, and cost-efficiency, the manufacturing process of bone constructs should ideally be compatible with the timeline of a single surgical procedure. In this case, tissue harvest, cell isolation, cell seeding onto a scaffold and subsequent implantation of a construct should occur within a few hours in an intra-operative manner, without ex vivo cell culture. In this perspective, stromal vascular fraction (SVF), the primary isolated cell population from a lipoaspirate, has more potential over ADMSCs.

SVF is a heterogeneous mixture of stromal cells, endothelial cells, pericytes, lymphocytes, mast cells, and pre-adipocytes [10–12]. Recent work has already demonstrated the feasibility of using this SVF for a one-step surgical approach to generate bone constructs [13, 14]. From preparation to implantation, this process can be finalized in few hours (less than 4 h) and has shown superior bone formation compared to cell-free scaffolds. To date, however, no scientific data report on the performance of SVF constructs compared to MSC-constructs, specifically for both types of constructs containing cells of the same human donor. Additionally, previous studies have shown beneficial effects of cell-cell interactions by applying multiple cell types in cell-based constructs [15, 16]. Other cells that can be easily isolated are monocytes (MO) from blood. Interestingly, monocytes have shown to stimulate osteogenic events by attracting MSCs and stimulating MSCs to proliferate and/or osteogenically differentiate [17, 18]. This observation justifies further translational research on the applicability of co-culture strategies using these two types of “easy access cells,” i.e., SVF and monocytes, to intraoperatively prepare bone constructs with superior osteogenic potential.

We here aimed to evaluate cellular interaction of human SVF and ADMSCs with human monocytes in vitro and their effects on bone regeneration in vivo. More importantly, we comparatively evaluate the bone regenerative efficiency of tissue-engineered constructs in an intraoperative manner (i.e., without in vitro expansion culture) and those in the conventional manner (i.e., with in vitro expansion culture).

## Methods

### Human cell isolation and culture

Human subcutaneous adipose tissue from three healthy donors (donor 1, female, 42 years old; donor 2, male, 38 years old; donor 3, female, 52 years old) was obtained from the Department of Plastic Surgery (Radboudumc) after ethical approval (Commissie Mensgebonden Onderzoek; dossier number #3252) and informed consent. The adipose tissue was excised into pieces and then minced in a 0.1% collagenase type II (Mannheim, Germany) solution with 1% bovine serum albumin (BSA, Sigma, St. Louis, USA) for 1 h at 37 °C under shaking conditions, as described previously [19]. After centrifugation at 600 g for 10 min at room temperature, the supernatant together with the fat layer was discarded. The resulting cell pellet was washed twice with phosphate-buffered solution (PBS; Gibco, Merelbeke, Belgium) with subsequent filtration through a polypropylene membrane with a pore size of 100 μm. After centrifugation, the cell pellet was suspended in proliferation medium, consisting of α-MEM (Gibco, Merelbeke, Belgium) containing 10% fetal bovine serum (FBS; Gibco, Carlsbad, USA). Red blood cells were lysed by incubation for 2 min in a solution of 7.49 g/L NH_4_Cl (2.06 g/L Tris Base, pH 7.2). Following centrifugation, the deposited cell aggregates were denoted as SVF, suspended in proliferation medium, and counted using a hemocytometer. This SVF was either frozen as 3 × 10^6^ cells per tube in the liquid nitrogen or used for further culturing to obtain a homogeneous population of ADMSCs.

For culturing, SVF containing 3 ×10^6^ cells was plated in 75-cm^2^ tissue culture flasks (Greiner Bio-one, Frickenhausen, Germany) in proliferation medium supplemented with 1 ng/ml of basic fibroblast growth factor (bFGF, Sigma-Aldrich, St. Louis, USA). Cells were grown in a humidified incubator at 37 °C in an atmosphere of 5% CO_2_. Two days after seeding, the adherent cells were thoroughly rinsed with PBS to remove non-adherent cells from the culture. In this way, a homogeneous population of ADMSCs was obtained. When reaching around 80% confluency, cells were detached with 0.05% trypsin in 0.5 mM EDTA (Sigma-Aldrich, St. Louis, USA) and were passaged. SVF and ADMSCs were characterized for surface marker expression by flow cytometry for the mesenchymal markers CD73 (APC-conjugated, BD Bioscience), CD90 (PE-Cy5-conjugated, BD Bioscience), CD105 (PE-conjugated, BD Bioscience, Franklin Lakes, USA), and negatively against CD45 (PE-conjugated, BD Bioscience). Osteogenic differentiation capacity of ADMSCs was confirmed by 4-week cultures in osteogenic medium (proliferation medium supplemented with 10 nM dexamethasone, 100 μM ascorbic acid, and 10 mM β-glycerophosphate).

The human monocytic cell line THP-1 was purchased from the American Type Culture Collection (ATCC; Manassas, USA) and cultured in RPMI-1640 medium (Gibco, Carlsbad, USA) supplemented with 10% FBS.

### Scaffold material for construct preparation

Tricalcium phosphate (TCP) granules were kindly provided by Cam Bioceramics (Leiden, the Netherlands). Pore sizes of TCP granules varied from 500 to 2 mm, and the granules had an overall porosity of 84%. After sterilization by gamma irradiation (SynergyHealth, Ede, the Netherlands), 11 mg (total volume around 21 mm^3^ corresponding to the bone defect size for in vivo experimental work) of these granules per sample were placed into individual 2-ml sterile Eppendorf tubes.

### Procedure for construct preparation

**Fig. 1:**
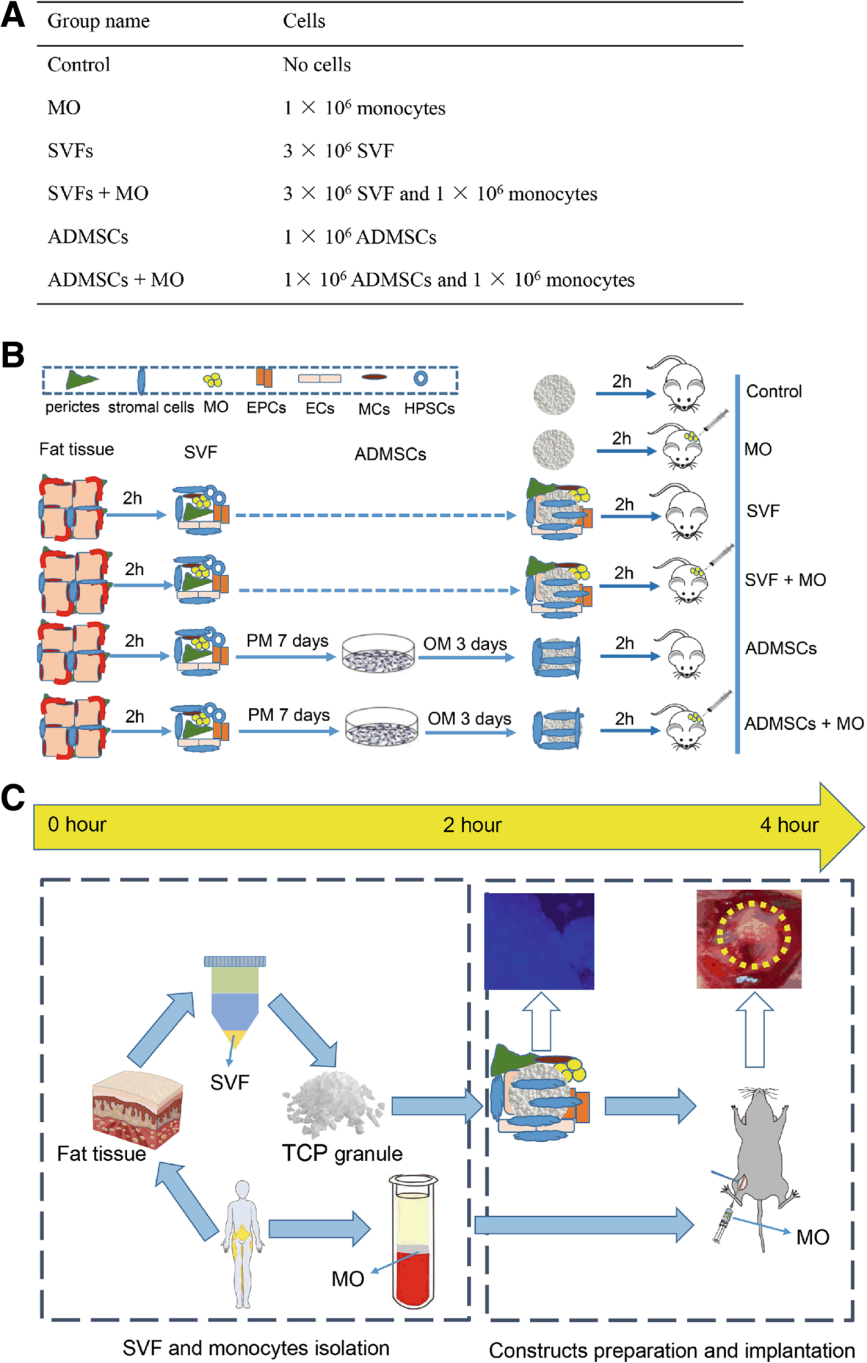
The preparation of intraoperative and conventional approach-based bone constructs. **a** Cell type and number used for different construct preparation. **b** Schematic structure of the experimental design. PM, proliferation medium; OM, osteogenic medium; EPCs, endothelial progenitor cells; ECs, endothelial cells; MCs, mast cells; HPSCs, hematopoietic stem/progenitor cells. **c** Timeline toward to the intraoperative preparation of cell-based bone constructs. The intraoperative bone constructs can be made within 4 h. Monocytes were added to the defect sites when bone constructs were put into femoral defects (yellow dashed circle)

Before construct preparation, 3 × 10^6^ SVF cells (cryo-preserved) were thawed, counted, and suspended in 300-μl proliferation medium to be loaded dropwise on top of TCP granules, which were pre-incubated in FBS for 24 h (hereafter referred to as “SVF groups”). The effective cryopreservation and retention of viability and differentiation capacity of SVF cells was previously shown [20]. The purified ADMSCs from SVF were expanded in proliferation medium with bFGF as described above. Osteogenic medium was applied for ADMSCs 3 days before preparation. 1 × 10^6^ ADMSCs were counted by a hemocytometer and suspended in 300-μl proliferation medium and were then loaded onto the granules (hereafter referred to as “ADMSCs groups”). All tubes were then supplemented with 300-μl proliferation medium and incubated at 37 °C for 2 h. Non-attached cells were collected and centrifuged before resuspension in the same medium and similarly dropwise loaded onto granules again. The process was repeated twice during this 2-h period. After that, cells with granules were collected for analysis or transferred into 24-well cell culture plates. Cell attachment was confirmed through 4′,6-diamidino-2-phenylindole (DAPI) staining and was quantified by DNA content quantification to ascertain comparable numbers of attached cells. After 2-h incubation, 1 × 10^6^ monocytes suspended in 200-μl proliferation medium were added to the SVF or ADMSCs constructs to make the SVF+MO and ADMSCs+MO groups. Same constructs were prepared for the animal study by directly adding monocytes to the defect sites during the surgery. TCP granules supplemented with the same volume of proliferation medium or 1 × 10^6^ monocytes only were set as the control and MO groups, respectively (Fig. 1a).

### In vitro assessment

#### Cell adhesion and viability on constructs

Cell attachment and cellular morphology on constructs were checked before implantation by identifying nuclei and actin of attached cells. The scaffolds were incubated in 20 μM Alexa Fluor® 568 Phalloidin (Thermofisher Scientific, Breda, Netherlands) for 15 min and in 300 nM DAPI stain for 5 min and then quantified with a Zeiss AxioPlan immunofluorescence microscope (Carl Zeiss Microimaging GmbH, Gottingen, Germany). Additionally, cell viability was assessed using a LIVE/DEAD® viability/cytotoxicity kit for mammalian cells (Invitrogen, Gaithersburg, MD, USA) according to the manufacturer’s instructions.

#### Cell proliferation and osteogenic differentiation on constructs

Constructs from the above six groups were cultured in osteogenic medium up to 4 weeks. After washing with PBS once, constructs were placed in 1 ml MilliQ and subjected to 2 cycles of freezing and thawing. Cell proliferation was evaluated by assessing the DNA content using a DNA quantification kit (Invitrogen, Carlsbad, CA) according to the manufacturer’s instructions. Osteogenic differentiation of all constructs was analyzed by quantification of alkaline phosphatase (ALP) activity of cell lysates using a 4-nitrophenyl phosphate-based method [19] after 1, 2, and 4 weeks of culture. To test mineralization, same type and number of cells without TCP granules were seeded in 24-wells cell culture plates. After 4 weeks, mineralization was evaluated by deposit calcium as described earlier [19]. Same cells cultured in the proliferation medium without osteogenic supplements were set in parallel as non-differentiation controls.

#### Effect of SVF or ADMSCs on macrophages polarization

To assess the effect of SVF or ADMSCs on macrophages polarization, 1 × 10^6^ ADMSCs or 3 × 10^6^ SVF were seeded on 11-mg TCP granules for 2 h as described above. 1 × 10^6^ THP-1 monocytes were seeded with 250 nM phorbol myristate acetate (PMA; monocytes were activated into macrophages) for 48 h in 24-well plates. These cell-loaded constructs and TCP granules were then transferred into 0.4-mm-pore size Corning transwell inserts (Sigma-Aldrich, Zwijndrecht, the Netherlands) and placed into the plates. Co-cultures were incubated for another 48 h. Wells supplemented with 100 ng/ml lipopolysachariden (LPS; Sigma-Aldrich, St Louis, MO, USA) and 20 ng/ml interferon-γ (IFN-γ; Sigma-Aldrich, St Louis, MO, USA) or 20 ng/ml interlukine-4 (IL-4; Sigma-Aldrich, St Louis, MO, USA) and 20 ng/ml interlukine-13 (IL-13; Sigma-Aldrich, St Louis, MO, USA) were set as control. The secreted tumor necrosis factor α (TNF-α) and transforming growth factor β (TGF-β) in the supernatants were evaluated using ELISA kits (e-Bioscience, San Diego, CA, USA) according to the manufacturer’s instructions.

### Animal surgery

The study was approved by the Centrale Commissie Dierproeven (CCD; project 2015-137); national guidelines for animal care and welfare were obeyed, conforming to the ARRIVE guidelines. A total of 46 nude rats (Crl:NIH-Foxn1^rnu^, Charles River) weighing between 250 and 300 g were used in the present study. All rats were pre-medicated with an intramuscular injection (5 mg/kg) of carprofen. General anesthesia with nitrous oxide, oxygen, and isoflurane were applied during the surgery. Bone defects were surgically created (3 mm diameter, 3 mm depth) bilaterally in the femoral condyles using a 3-mm burr (Hager & Meisinger GmbH, Neuss, Germany) at low rational speed. MO (*n* = 14), SVF (*n* = 16), SVF (*n* = 16), ADMSCs (*n* = 16), and ADMSCs+MO (*n* = 16) grafted implants were then placed into defects with acellular controls (*n* = 14). The soft tissue incision was then closed with superficial sutures. In all cases, the transplantation was performed within 4 h. After surgery, 150 mg/kg buprenorphine (Temgesic, Schering-plough, Amstelveen, the Netherlands) was subcutaneously given for 2 days to reduce postoperative pain. Four and 10 weeks after implantation, all rats were sacrificed by inhalation of CO_2_. The constructs with surrounding tissue were harvested and fixed in a 4% formalin buffer for 48 h. Samples were randomly selected and then dehydrated and embedded in paraffin for histochemistry and immunohistochemistry process. The rest of samples were randomly distributed, dehydrated, and embedded in polymethyl methacrylate (PMMA) for the histological process.

### Histological and histomorphometric analysis

After fixation, certain specimens were dehydrated in a graded series of ethanol and embedded in PMMA without decalcification. Specimens were sectioned longitudinally (335 μm thick) using a diamond saw (Leica Microsystems SP 1600, Nussloch, Germany). Three sections representative of the center of the defect and subcutaneous sample from each specimen were then stained with methylene blue/basic fuchsin to evaluate new bone formation. Stained samples were further photographed with Zeiss Imager Z1 microscope equipped with the Axiocam camera using AxioVision 4.8 software. After that, the bone formation percentage (BF) was determined in stained sections using an automated image-analysis system (Leica Qwin Proimage analysis system, Wetzlar, Germany) which recognize bone tissue based on different RGB values from highly magnified images. Minor manual corrections were made to ensure the precise selection of newly formed bone in the defects. The percentage of new bone formation was measured by normalized to initial defect area measured from the cross section.

### Immunohistochemical and histochemical staining

After fixation, samples from each group were decalcified in an EDTA solution, dehydrated in a series of alcohol and embedded in paraffin. The specimens were sectioned (*n* = 3) at a thickness of 5 μm and stained with hematoxylin and eosin for conventional, qualitative bright field light microscopy analysis. Three additional sections per specimen were further stained with Masson’s trichrome and observed by light microscopy. For immunostaining, paraffin sections were rehydrated in serials of ethanol and antigen was retrieved in sodium citrate buffer (PH 6.0) at 70 °C for 10 min. Subsequently, slides were blocked with 10% normal donkey serum (NDS) and then incubated with the primary antibody overnight at 4 °C. Slides were then treated with a biotin-conjugated secondary antibody (Chemicon, Temecula, USA) for 1 h at room temperature, followed by counterstaining with hematoxylin. Negative controls using 2% NDS instead of the primary antibody were generated in parallel to ensure that the staining was specific. Finally, the sections were dehydrated and mounted. Stained sections were photographed with the same Zeiss Imager Z1 microscope. To detect human cells in the constructs implanted in rats, immunostaining was performed using human specific anti-mitochondria antibody (EMD Millipore, No. MAB1273, Amsterdam-Zuidoost; diluted 1:200) as protocol indicated. The number of human cells per construct was calculated by volumetrically relating slide volume to defect volume. Human and rat skin sections were used as positive and negative controls, respectively. The presence of human origin macrophages was assessed by using human specific mouse anti-human CD68 (Dako, Leuven, Belgium; diluted 1:200). To stain blood vessles, monoclonal antibody mouse anti- human/rat α-smooth muscle actin (α-SMA, Sigma-Aldrich, No. A2547, Billerica, USA; diluted 1:128,000) was used. For Tartrate-resistant acid phosphatase (TRAP) staining, sections were deparaffinized, rinsed in PBS and incubated with a solution containing 50 mM sodium acetate (pH 5.2), 0.15% Naphtol-AS-TR-phosphate, 50 mM sodium tartrate, and 0.1% Fast Red T.R. (Sigma-Aldrich Chemie Gmbh, Taufkirchen, Germany) for 30–40 min at room temperature. Subsequently, the sections were rinsed in PBS and counterstained.

### Statistical analysis

Cells from three donors were used for in vitro experiments and cells from donor 1 were used for in vivo donor-matched comparison. Data are presented as means ± standard deviations (SD). Statistical analysis was performed using a one-way ANOVA with Graphpad Prism 5 software. When ANOVA indicated a significant difference between different groups, a Tukey Post-hoc Multiple Comparisons was performed. An unpaired Student’s *t* test was used to compare the calcium content between SVF and ADMSCs. *P* values < 0.05 were regarded as significant.

## Results

### Comparative characterization of human ADMSCs and SVF

Before construct preparation, we performed cytofluorimetric analysis to characterize SVF and ADMSCs respectively. The analysis of stromal cell markers (CD73, CD90, and CD105) showed consistent presence of stromal cells in SVF and stromal cells took up around one third of the SVF population (Additional file 1: Figure S1).

### Preparation of constructs and viability assessment

To prepare SVF constructs, we seeded 3 × 10^6^ SVF cells on 21 mm^3^ TCP granules and incubated these in proliferation medium for 2 h to allow for cell attachment. Similarly, we seeded 1 × 10^6^ ADMSCs on TCP granules to obtain a comparable number of stromal cells on each construct. Subsequently, we added 1 × 10^6^ monocytes to the SVF and ADMSC constructs in wells in vitro or to the constructs in the defects in vivo (Fig. 1a). Based on the design, from the isolation of SVF cells and peripheral blood monocytes till implantation of SVF constructs with monocytes, this procedure can be performed within 4 h (Fig. 1b, c). In contrast, the conventional ADMSC-based approach takes at least 10 days. To assess cell attachment to the prepared constructs, we performed nuclei and actin staining. Cells showed homogeneous distribution over the surface of granules (Additional file 2: Figure S2). Cell viability after 2 h in vitro incubation demonstrated that the majority of cells attached to the granules were viable, without apparent differences in dead cells between the experimental groups (Additional file 2: Figure S2).

### Monocytes promote osteogenic differentiation of SVF and ADMSCs

**Fig. 2:**
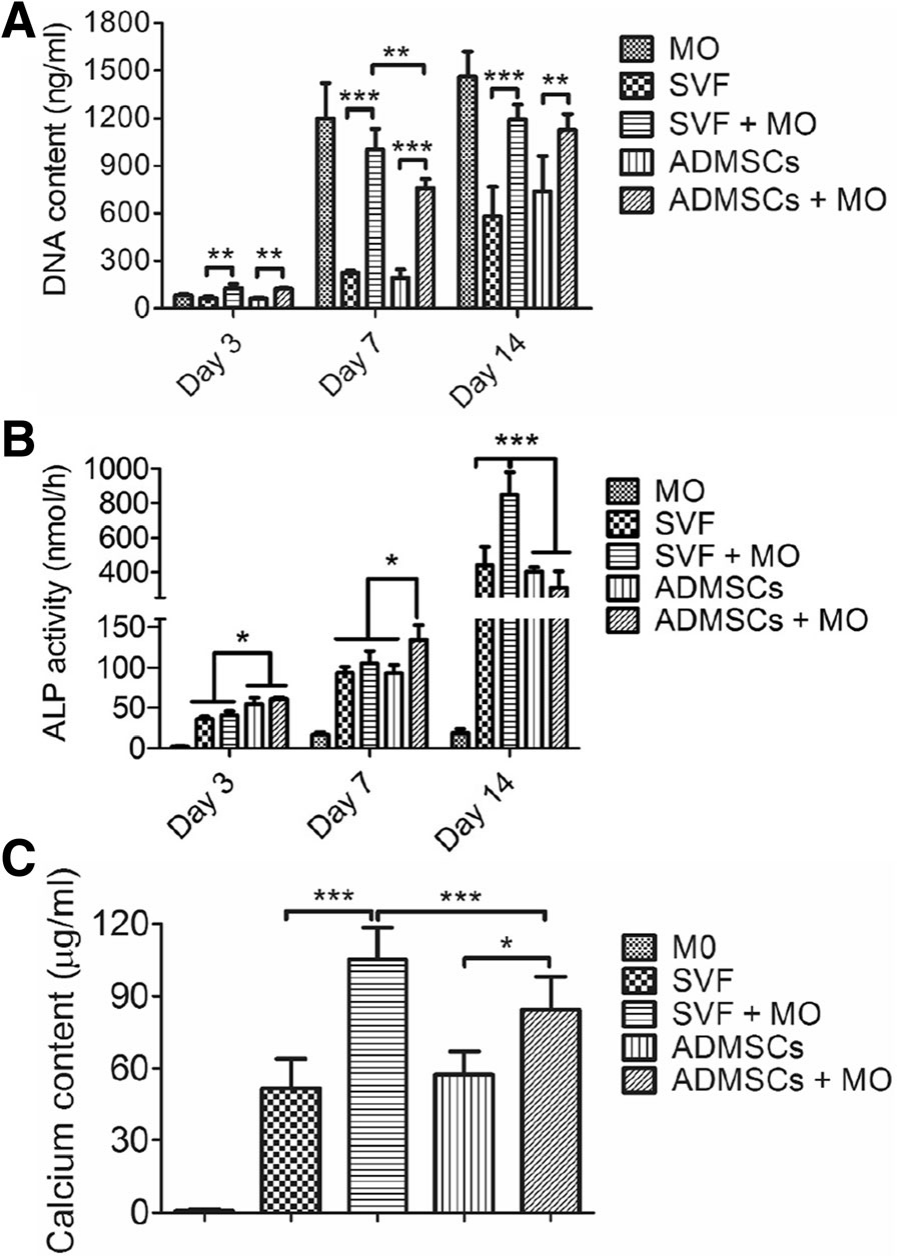
In vitro cell behavior of SVF and ADMSCs upon co-culture with monocytes*.* **a** Cell proliferation of SVF, ADMSCs, and co-culture with monocytes quantified by DNA content (*n* = 4; *** < 0.001). **b** Osteogenic differentiation of SVF, ADMSCs, and co-culture with monocytes quantified by ALP activity (*n* = 4; * < 0.05; *** < 0.001). **c** Mineralization of SVF, ADMSCs, and co-culture with monocytes after 4 weeks quantified by calcium content (*n* = 4; * < 0.05; *** < 0.001)

To study cellular behavior upon effect of cell-cell interactions between monocytes and SVF or ADMSCs, we cultured cell-loaded constructs in osteogenic medium for up to 4 weeks and assessed cell proliferation (DNA content; Fig. 2a), osteogenic differentiation (ALP-activity; Fig. 2b), and mineralization (calcium assay; Fig. 2c). All cells on the granules showed proliferation over time. More specific, SVF and ADMSCs showed similar cell proliferation profiles (Fig. 2a). In contrast, cultures containing monocytes showed a significantly increased proliferation (Fig. 2a). Additionally, whilst monocyte monocultures did not show ALP activity, monocytes slightly increased ALP activity in co-cultures with either SVF or ADMSCs (Fig. 2b). Moreover, monocytes further significantly increased mineralization in co-cultures with either SVF (~ 2-fold) or ADMSCs (~ 1.5-fold) compared to SVF and ADMSCs monocultures in osteogenic medium, respectively (Fig. 2c). In contrast, all cultures after seeding (day 0) and constructs cultured in proliferation medium (non-differentiation controls) had a negligible value for ALP activity and calcium content (data not shown).

### SVF cells and ADMSCs induce distinct polarization of monocytes/macrophages

**Fig. 3:**
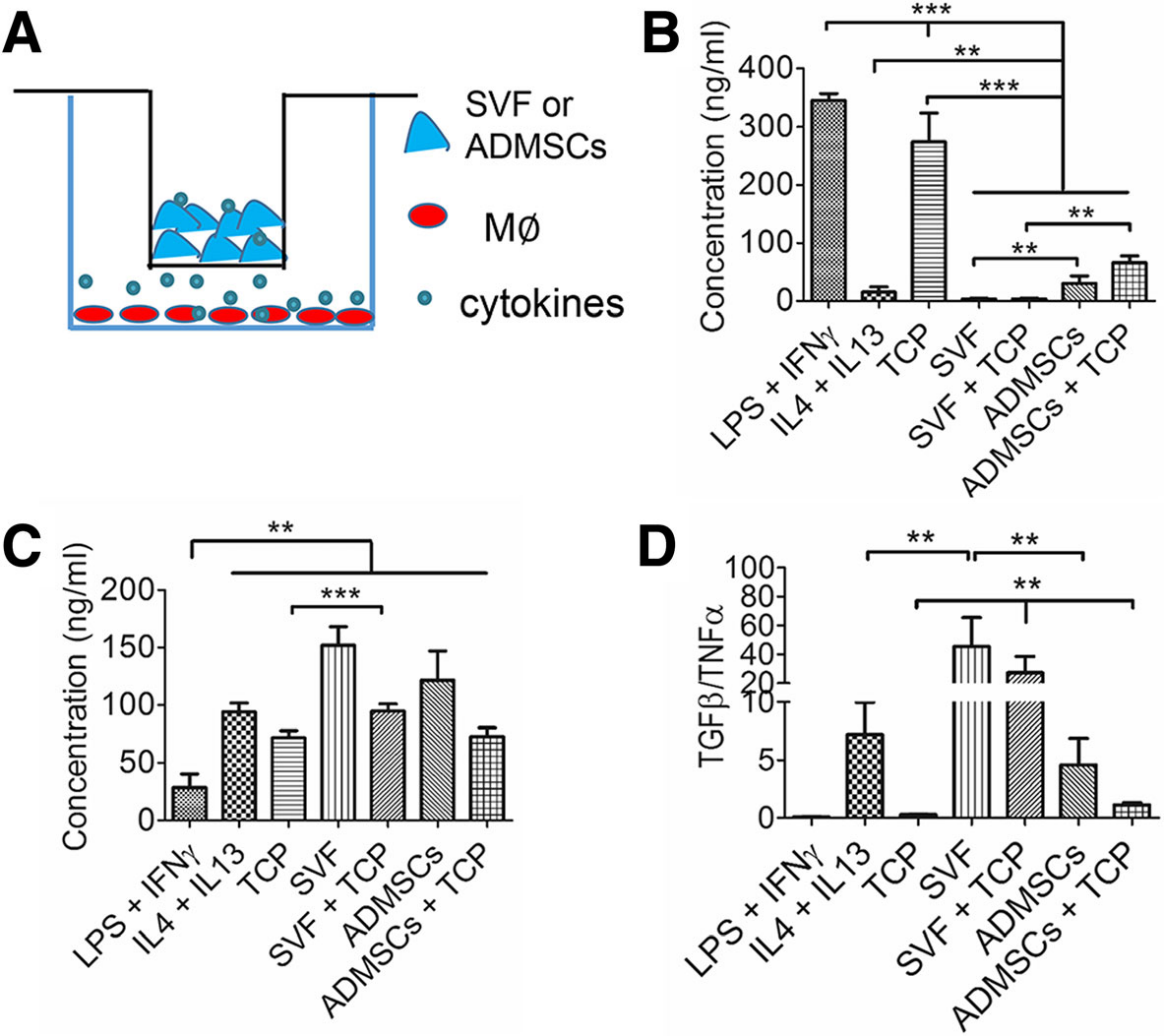
In vitro cell behavior of monocytes upon co-culture with SVF or ADMSCs. **a** Schematic diagram of SVF and ADMSCs indirectly co-cultured with macrophages by a transwell. **b** Quantification of pro-inflammatory cytokine TNF-α in the co-culture medium (*n* = 6; **p < 0.01; *** < 0.001). **c** Quantification of anti-inflammatory cytokine TGF-β in the co-culture medium (*n* = 6; **p < 0.01; *** < 0.001). **d** Ratio of TGF-β/TNF-α **(***n* = 6; **p < 0.01). LPS- and IFN-γ-induced M1 macrophages and IL-4- and IL-13-induced M2 macrophages were set as control respectively

In parallel with our experiments focused on the effect of monocyte addition to either SVF or ADMSC cultures, we oppositely focused on effects of SVF or ADMSCs on monocytes activation and polarization. Therefore, we set up an in vitro culture model [21, 22] and analyzed macrophage polarization via cytokine expression profiles of the pro-inflammatory M1 macrophage cytokine TNF-α and the pro-wound healing M2 macrophage cytokine TGF-β (Fig. 3a). Conventional induction into M1 macrophage polarization via LPS+IFN-γ and into M2 macrophage polarization via IL-4+IL-13 stimulation was used as control. TCP granules used as cell carrier material in our experiments induced polarization into a pro-inflammatory M1 macrophage, while SVF and ADMSCs induced macrophage polarization toward the pro-wound healing M2 type (Fig. 3b, c). The ratio TGF-β/TNF-α (Fig. 3d) clearly shows the overall M2 macrophage polarization stimulation, particularly by SVF.

### Cell-based constructs enhance early bone healing

**Fig. 4:**
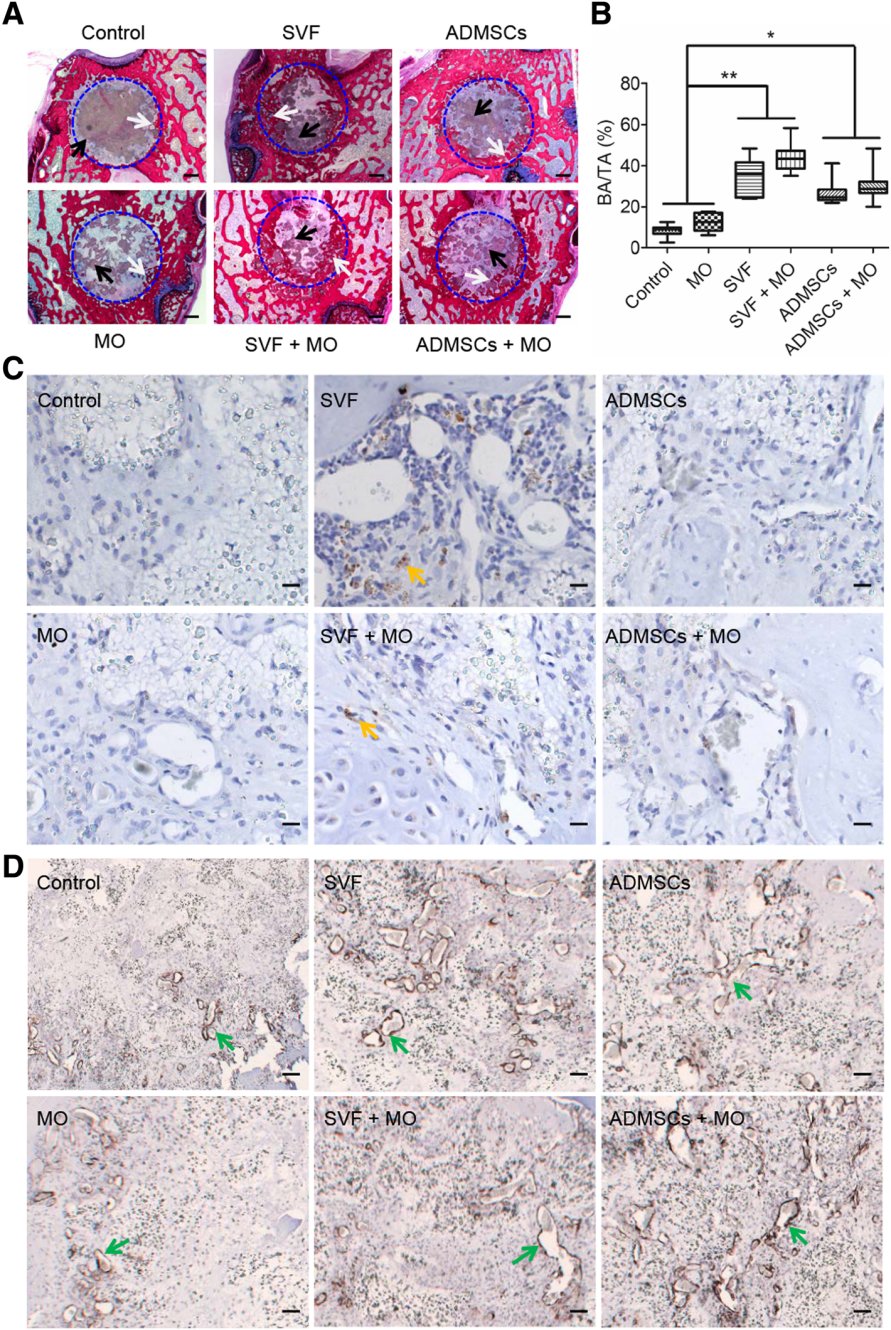
New bone quantification and representative images of histology and immunohistochemistry staining after 4 weeks implantation in the femoral condyle bone defect. **a** Representative histological sections (methylene blue/basic fuchsin). Blue dashed circles indicate original bone defect, black arrows indicate TCP granulate, and white arrows indicate newly formed bone; scale bar = 500 μm. **b** Histomorphometric analysis of new bone area (BA) within the total defect area (TA; *n* = 6 to 7; *p < 0.05; ** < 0.01). **c** Representative images of anti-human mitochondria staining. Yellow arrows indicate positive staining. **d** Representative images of anti-α-SMA staining. Green arrows indicate blood vessels; scale bar = 10 μm

To assess the bone regeneration capacity, we implanted control, MO, SVF, SVF+MO, ADMSCs, and ADMSCs+MO constructs (*n* ≥ 7 for each group) into rat femoral defects (3 mm in diameter, 2.8 mm in depth) and evaluated bone regeneration histologically and histomorphometically after 4 and 10 weeks. Methylene blue/basic fuchsin staining (Fig. 4a) displayed substantial amounts of newly formed bone at the defect margins in between the voids of TCP granules in all SVF and ADMSC constructs after 4 weeks, irrespective of monocyte addition. In contrast, control and MO constructs showed limited amounts of newly formed bone. Histomorphometrically, SVF (34.5 ± 8.6%) showed similar amounts of new bone formation to ADMSCs (27.1 ± 6.0%), both of which regenerated approximately three times more bone compared to control and MO (Fig. 4b).

We further assessed the contribution of SVF and ADMSCs to bone formation using specific anti-human mitochondrial staining to detect human cells (i.e., SVF, ADMSCs and monocytes) within the bone defect area (Fig. 4c and Additional file 3: Figure S3). SVF and SVF+MO constructs clearly showed the presence of human cells (17,800 ± 19,530 and 5600 ± 6460 per construct respectively) in the vicinity of the TCP granulate and newly formed bone, but not within the newly formed bone. This positive staining for human cells originated from SVF, as we observed no positive staining for the human monocyte/macrophage marker CD68 (Additional file 4: Figure S4) nor for the osteoclastic marker TRAP (Additional file 5: Figure S5). In contrast, ADMSCs and ADMSCs+MO constructs did not show any human cells within histological sections. In addition, SVF and ADMSCs constructs showed apparently larger vessel-like structures compared to control and MO constructs, which suggests beneficial effects of SVF and ADMSCs on the vascularization of implanted constructs (Fig. 4d).

### SVF constructs show complete bone defect healing

**Fig. 5:**
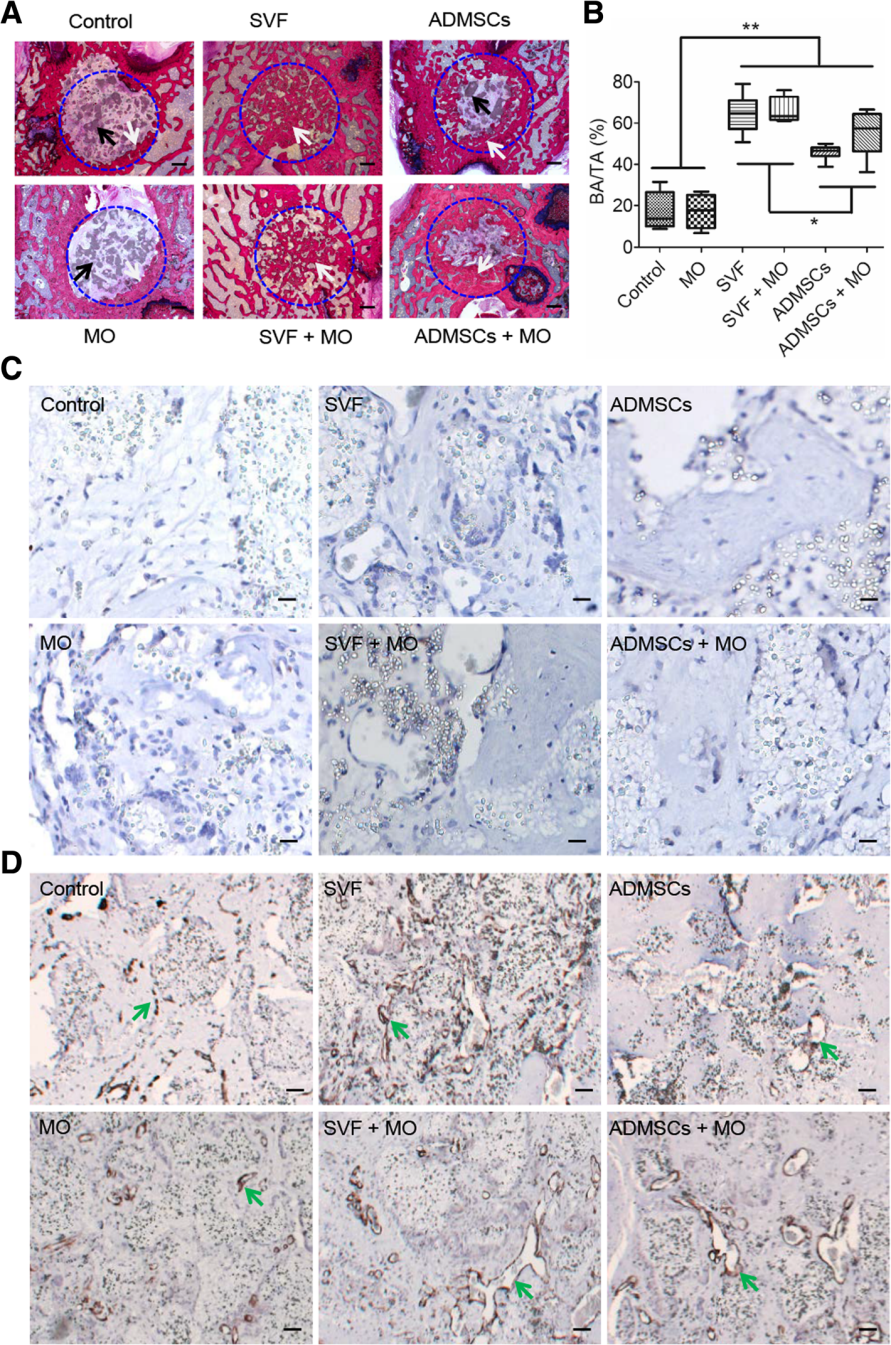
New bone quantification and representative images of histology and immunohistochemistry staining after 10 weeks implantation in the femoral condyle bone defect. **a** Representative histological sections (methylene blue/basic fuchsin). Blue dashed circle indicate original bone defects, black arrows indicate TCP granulate, and white arrows indicate newly formed bone; scale bar = 500 μm. **b** Histomorphometric analysis of new bone area (BA) within the total defect area (TA; *n* = 6 to 7; *p < 0.05; ** < 0.01). **c** Representative images of anti-human mitochondria staining. Yellow arrows indicate positive staining. **d** Representative images of anti-α-SMA staining. Green arrows indicate blood vessels; scale bar = 10 μm

At 10 weeks post implantation, SVF constructs (w/- MO) exhibited complete healing of the defect site with newly formed bone present throughout the entire defect area (Fig. 5a). In contrast, ADMSCs (w/- MO) showed substantial bone ingrowth centripetally from the original defect edge toward the defect center without presence of newly formed bone in the center. Control and MO constructs still showed large defect areas containing fibrous connective tissue with only sparse bone formation at the edge of the original defect. Residual TCP granulate was still present in non-healing defects and integrated with new bone in SVF and ADMSCs constructs. Quantitative analysis of new bone formation by histomorphometry (Fig. 5b) revealed superior amounts of newly form bone for SVF and SVF+MO constructs (64.9 ± 9.1% and 66.3 ± 6.0%, respectively) compared to ADMSCs (45.4 ± 3.7%), ADMSCs+MO (55.2 ± 10.6%), MO (17.3 ± 8.0%), and Control (17.2 ± 9.0%) constructs. Further, ADMSC and ADMSC+MO constructs showed significantly higher bone formation compared to MO and control constructs.

Anti-human mitochondrial staining to detect cells of human origin demonstrated absence of human cells for any type of implanted construct after 10 weeks (Fig. 5c). Based on α-SMA staining to assess vascularization, constructs with SVF or ADMSCs apparently gained more organized and elongated vessel-like structures (Fig. 5d). Different to our observations after 4 weeks of implantation, osteoclastic activity was abundantly present in all defects containing cell-based constructs and only to a limited extent in control constructs (Additional file 5: Figure S5).

## Discussion

Cell-based bone tissue engineering, especially with the application of bone marrow-derived MSCs, represents an appealing alternative for the current best practice treatments using auto-/allografts for bone regeneration. However, this approach is challenged by complex, impractical and expensive in vitro cell expansion due to the low numbers of MSCs in the bone marrow. Aiming to circumvent these, we followed an intraoperative approach based on stromal vascular fraction (SVF) from fat tissue, as described recently [13, 14]. However, the efficacy of this intraoperative approach has not been directly compared to conventional ADMSC-based method with cells of the same donor. As another type of easy access cells with potential stimulatory effects on wound healing and stromal cell differentiation [17, 18], we explored the addition of monocytes to both ADMSC and SVF constructs both in vitro and in vivo using a rat femoral condyle bone defect model.

SVF is reported to be a heterogeneous mixture of MSCs, endothelial progenitor cells, pericytes, mast cells, pre-adipocytes, and other cell types [7, 11]. From flow cytometry and in vitro cell culture results, we proved that MSCs take up around one third of the whole SVF population, which corroborates previous reports [23–25]. As MSCs were designated as the main effective cells in bone formation, we used 3 million SVF cells versus 1 million purified ADMSCs, which were isolated from the same donor during the same procedure. We then compared their osteogenic potential in vitro and bone regeneration capacity in vivo. In vitro mineralization demonstrated a similar osteogenic potential between SVF and ADMSCs. In contrast, although both SVF and ADMSCs improved bone defect healing compared to cell-free controls, data from our in vivo work demonstrated superiority of SVF constructs over ADMSC constructs regarding bone-regenerative capacity. This difference between in vitro and in vivo results suggests that in vitro osteogenic differentiation capacity is not predictive for in vivo bone healing capacity. The synergetic effect of endothelial progenitor cells and pericytes contained in SVF probably plays an important role in the superiority of SVF bone healing [24, 26]. Important observations, likely related to the superior bone regeneration for SVF and SVF + MO constructs, were the prolonged presence of SVF cells in the bone defect region compared to ADMSCs in spite of in low numbers (less than 2% of seeded cells in the constructs after 4 weeks implantation). The lower number of human cells in SVF+MO compared to SVF likely resulted from the low survival and proliferation rates of seeded SVF cells subjected to more stringent inflammation due to the addition of monocytes [27–29].

Speculating about the mechanism of SVF- and ADMSCs-based bone regeneration, our results and those of others [30] indicate that direct differentiation of grafted cells into bone forming osteoblasts or eventually embedded osteocytes seems very unlikely. Further, it has been postulated that the grafted cells provide osteoinductive signals or act as a source of trophic factors to modulate microenvironments [31–33]. Here, we did not observe grafted human cells within the newly formed bone (only in the vicinity at 4 weeks), ruling out the mechanism of direct differentiation of SVF or ADMSCs into bone forming osteoblasts or osteocytes. Consequently, we speculate that SVF and ADMSCs exerted a certain paracrine effect on surrounding host cells to stimulate bone regeneration. The exact paracrine factors secreted by these implanted cells responsible for bone healing are largely unknown yet due to the complexity of in vivo conditions. Still, few pioneering reports indicated several possible roles of grafted cells, such as promoting progenitor cells migration [34, 35], benefiting vascularization [31, 36], and modulating immune response [37, 38], to stimulate bone regeneration. Amongst these hypotheses, the immunomodulatory role by MSCs in tissue regeneration recently gained much attention. MSCs have been shown to modulate the function of both innate and adaptive immune cells through direct interactions as well as releasing numerous bioactive soluble factors [39, 40]. In particular, MSCs can directly polarize naïve macrophages toward the M2 phenotype to exert a therapeutic effect on skin, brain, and muscle [41–43]. In bone regeneration process, M2 phenotype macrophages can promote tissue remodeling by releasing cytokines such as basic fibroblast growth factor (bFGF) to benefit the host vascularization [44] and increase osteogenesis by releasing certain growth factors such as OSM and BMP-2 [45]. In the present study, SVF and ADMSCs activated and polarized monocytes/macrophages into M2 phenotype as proved in in vitro co-culture experiment. These M2 macrophages likely further led to more profound vascularization as our results and previous studies reported [24] and subsequent more bone formation compared to constructs without SVF and ADMSCs in vivo. The coincidence of superior immunoregulatory effect and more remarkable bone regeneration capacity by grafted SVF further support the hypothesis about immunoregulatory roles in bone healing. Nonetheless, this warrants further investigations into the paracrine factors underlying, such as recently reported extracellular vesicles route [46, 47].

Due to their reported beneficial effects on osteogenic differentiation [17, 18] and the easy access via a venous puncture, the concept of harnessing the power of monocytes/macrophages for bone tissue engineering was newly proposed [48, 49]. However, it has never been applied for cell-based bone regeneration. Our study also confirmed their beneficial effects on in vitro osteogenic differentiation, evidenced by significantly higher ALP activity and mineralization in co-cultures with SVF or ADMSCs. Interestingly, monocytes exerted a more obvious effect on SVF compared to ADMSCs, which suggests differences in cell status (undifferentiated versus differentiated), cell components (heterogeneous and homogenous), and cell-cell interactions of SVF compared to ADMSCs. Nonetheless, in our orthotopic animal model, we did not observe this favorable effect on bone regeneration following monocyte addition. As we did not observe specific immunostaining for human monocytes/macrophages in any of our histological sections (Additional file 3: Figure S3), this might be explained by the short duration of exogenously added monocytes present in the defects, the relatively limited half-live of this cell life, and less adhesive properties compared to stromal cells [50, 51]. However, given the newly reported pivotal roles of immune cells, including monocytes, macrophages, NK cells, and lymphocytes in tissue regeneration [49, 52, 53], introduction of immune cells in cell-based bone tissue engineering still seems an attractive treatment strategy for bone regeneration.

Our study also has several limitations. First, we used human monocytes from a cell line rather than human primary monocytes considering the experimental feasibility and reproducibility. Although THP-1 has been proven as a suitable cell culture model for primary monocytes [54], further studies are needed to analyze the behavior of SVF and ADMSCs co-cultured with primary monocytes, especially when using monocytes and SVF derived from the same patient. Secondly, SVF and ADMSC constructs enhanced bone formation after 4 weeks, which suggests that the seeded cells function in the early post-implantation stage. Therefore, observation of host response (i.e., cell infiltration, vascularization, and macrophage phenotype) at earlier time points would likely provide more clarity on the potential immunoregulatory role of grafted cells in bone healing. In addition, due to the component similarity and close distribution between TCP and newly formed bone, it is not practical to use regular X-ray or micro-CT to provide additional information on bone density and quality.

Above all, instead of merely focusing on the outcome of cell-based bone tissue engineering as in most studies, we emphasized particularly on the potential of cell-based constructs for clinical applications. Since both SVF and monocytes can be freshly isolated via standard procedures during the surgical procedure, this strategy meets the requirements of “good manufacturing practice” and “during the same visit” defined by the concept of intraoperative preparation. In addition, this method likely reduces the high-cost associated to ex vivo cell expansion and its safety was confirmed in various clinical applications [13, 55]. Therefore, this ‘easy access cells’ based intraoperative approach and its superior bone-regenerative capacity will greatly aid the concept, design, development, and practicality of cell-based bone tissue engineering.

## Conclusion

To conclude, this study described the feasibility of a clinically compliant method to intraoperatively generate bone constructs by using easy access cells, namely stromal vascular fraction (SVF) from adipose tissue and monocytes from the peripheral blood. Our data demonstrated the equal osteogenic potential of SVF as expanded ADMSCs in vitro, which for both was significantly enhanced upon co-culture with monocytes. When applied to bone defects, SVF demonstrated a significantly higher bone formation compared to constructs grafted with ADMSCs and cell-free controls. This was further indicated attributing to the distinct differences of immunoregulatory effects between SVF and ADMSCs on macrophages. Given the superior bone regeneration capacity of SVF and the easy access of SVF and monocytes during the surgical procedure, the strategy proposed in this study has great potential in the clinic to develop new cell-based bone products to treat damaged bone.

## Additional files

Additional file 1: **Figure S1.** Cell characterization of SVF and ADMSCs by flow cytometry. SVF and ADMSCs were stained with APC-conjugated CD73, PE-Cy5-conjugated CD90, PE-conjugated CD105 and FITC-conjugated CD45 and then sorted by flow cytometry. (TIF 7284 kb)

Additional file 2: **Figure S2.** Cell attachment and viability on TCP granules after seeding for 2 h. For the attachment, cells were stained with DAPI for the nuclei (blue) and Alexa Fluor 568 conjuncted phalloidin for the actin (red); scale bar = 500 μm. For the viability, cells were stained with calcein-AM (green) and ethidium homodimer-1 (red). White arrows indicate live cells and red arrows indicate dead cells; scale bar = 500 μm. (TIF 10203 kb)

Additional file 3: **Figure S3.** Representative images of anti-human mitochondria staining after 4 weeks orthotopic implantation. (A) SVF constructs at low magnification; Scale bar = 500 μm. (B) SVF constructs at high magnification. NB indicates new bone and TCP indicates TCP granules. Brown arrows indicate human origin cells. Scale bar = 100 μm. (C) SVF+MO constructs at low magnification; Scale bar = 500 μm. (D) SVF+MO constructs at high magnification. NB indicates new bone and TCP indicates TCP granules. Brown arrows indicate human origin cells; scale bar = 100 μm. (TIF 5613 kb)

Additional file 4: **Figure S4.** Representative images of anti-human CD68 immunohistochemistry staining after 4 weeks orthotopic implantation. Black arrow indicates TCP granules. Yellow arrow indicates presence of human macrophages in the samples. PC indicates the positive control samples stained with anti-human CD68; Scale bar = 100 μm. (TIF 3236 kb)

Additional file 5: **Figure S5.** Representative images of TRAP immunohistochemistry staining after (A) 4 and (B) 10 weeks orthotopic implantation. Blue arrows indicate TRAP-positive signals in the defect region; bar = 500 μm. (TIF 9162 kb)

## Abbreviations

ADMSCs: Adipose tissue mesenchymal stem cells
SVF: Stromal vascular fraction
MSCs: Mesenchymal stromal cells
MO: Monocytes
TRAP: Tartrate-resistant acid phosphatase
TCP: Tricalcium phosphate
BSA: Bovine serum albumin
PMA: Phorbol myristate acetate
LPS: Lipopolysachariden
IL-4: Interlukine-4
IL-13: Interlukine-13
IFN-γ: Interferon-γ
TNF-α: Tumor necrosis factor-α
TGF-β: Transforming growth factor-β
ALP: Alkaline phosphatase
bFGF: Basic fibroblast growth factor
DAPI: 4′,6-Diamidino-2-phenylindole
