# Supplementary figures and images for: Efficacy of intraoperatively prepared cell-based constructs for bone regeneration
Source: Stem Cell Res Ther. 2018 Oct 25;9:283. doi: 10.1186/s13287-018-1026-7 (PMC6203202; doi:10.1186/s13287-018-1026-7)

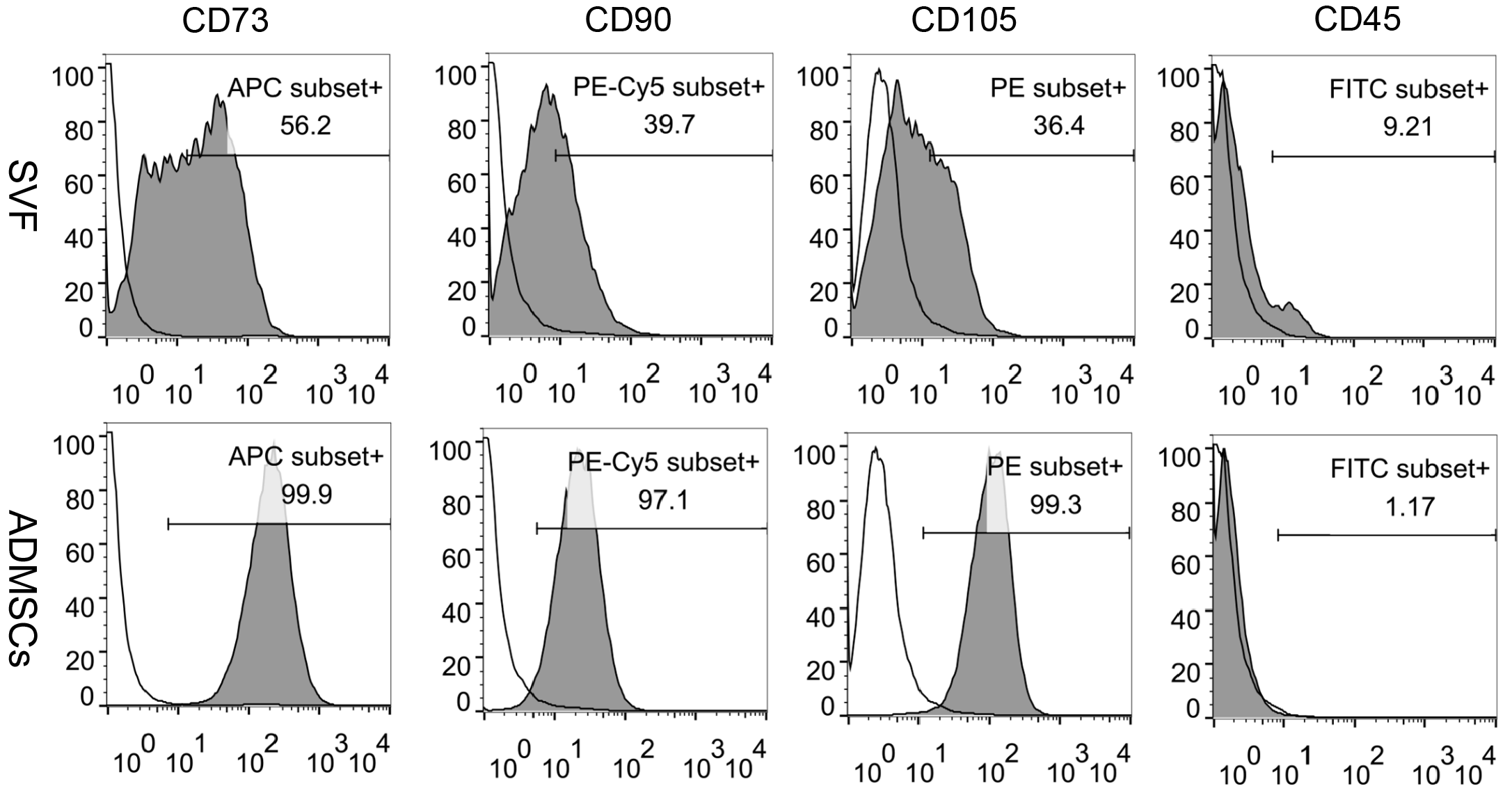

Supplement: Supplementary file 1 — Figure S1. Cell characterization of SVF and ADMSCs by flow cytometry. SVF and ADMSCs were stained with APC-conjugated CD73, PE-Cy5-conjugated CD90, PE-conjugated CD105 and FITC-conjugated CD45 and then sorted by flow cytometry. (TIF 7284 kb) [file 13287_2018_1026_MOESM1_ESM.tif]

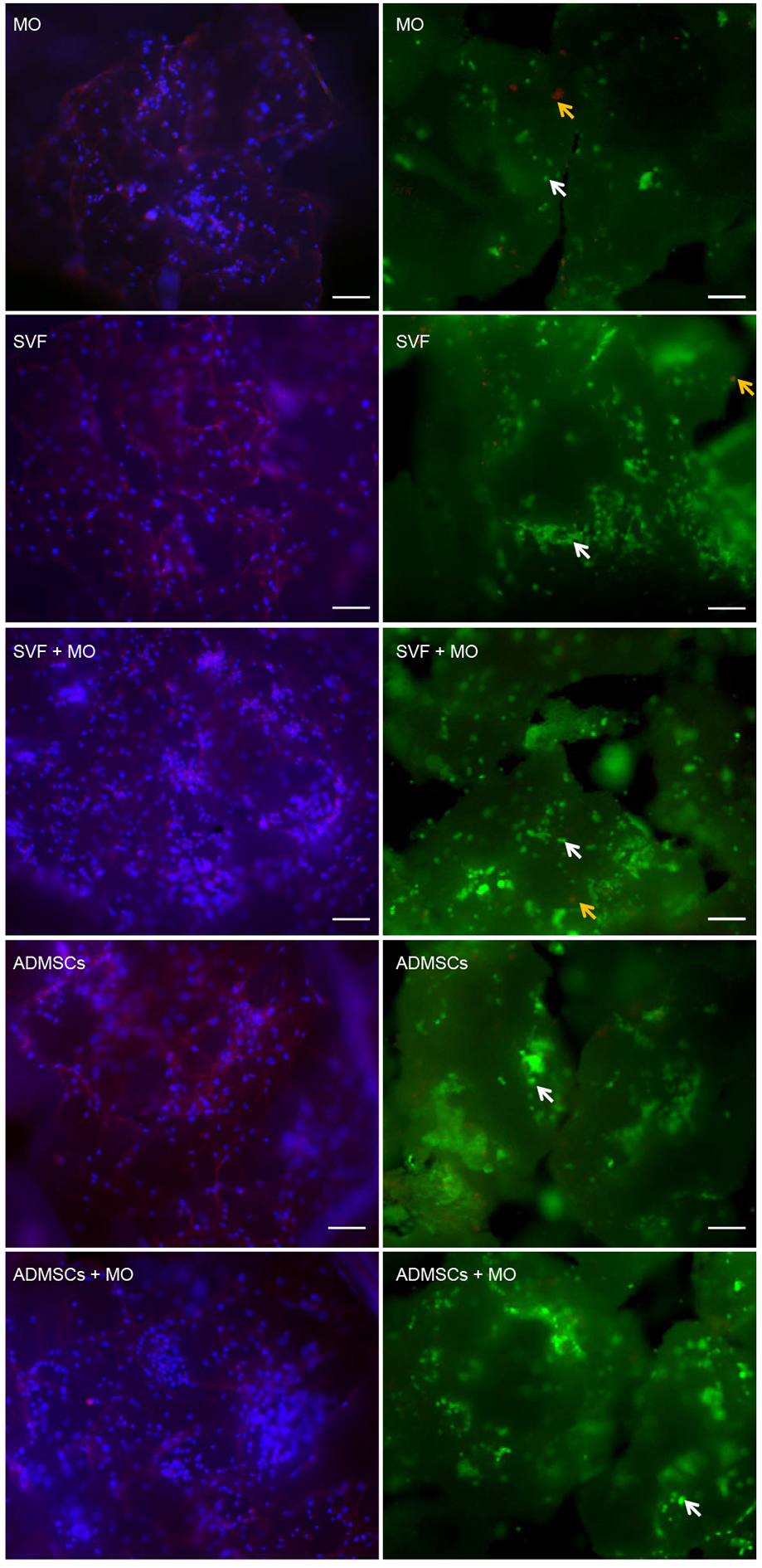

Supplement: Supplementary file 2 — Figure S2. Cell attachment and viability on TCP granules after seeding for 2 h. For the attachment, cells were stained with DAPI for the nuclei (blue) and Alexa Fluor 568 conjuncted phalloidin for the actin (red); scale bar = 500 μm. For the viability, cells were stained with calcein-AM (green) and ethidium homodimer-1 (red). White arrows indicate live cells and red arrows indicate dead cells; scale bar = 500 μm. (TIF 10203 kb) [file 13287_2018_1026_MOESM2_ESM.tif]

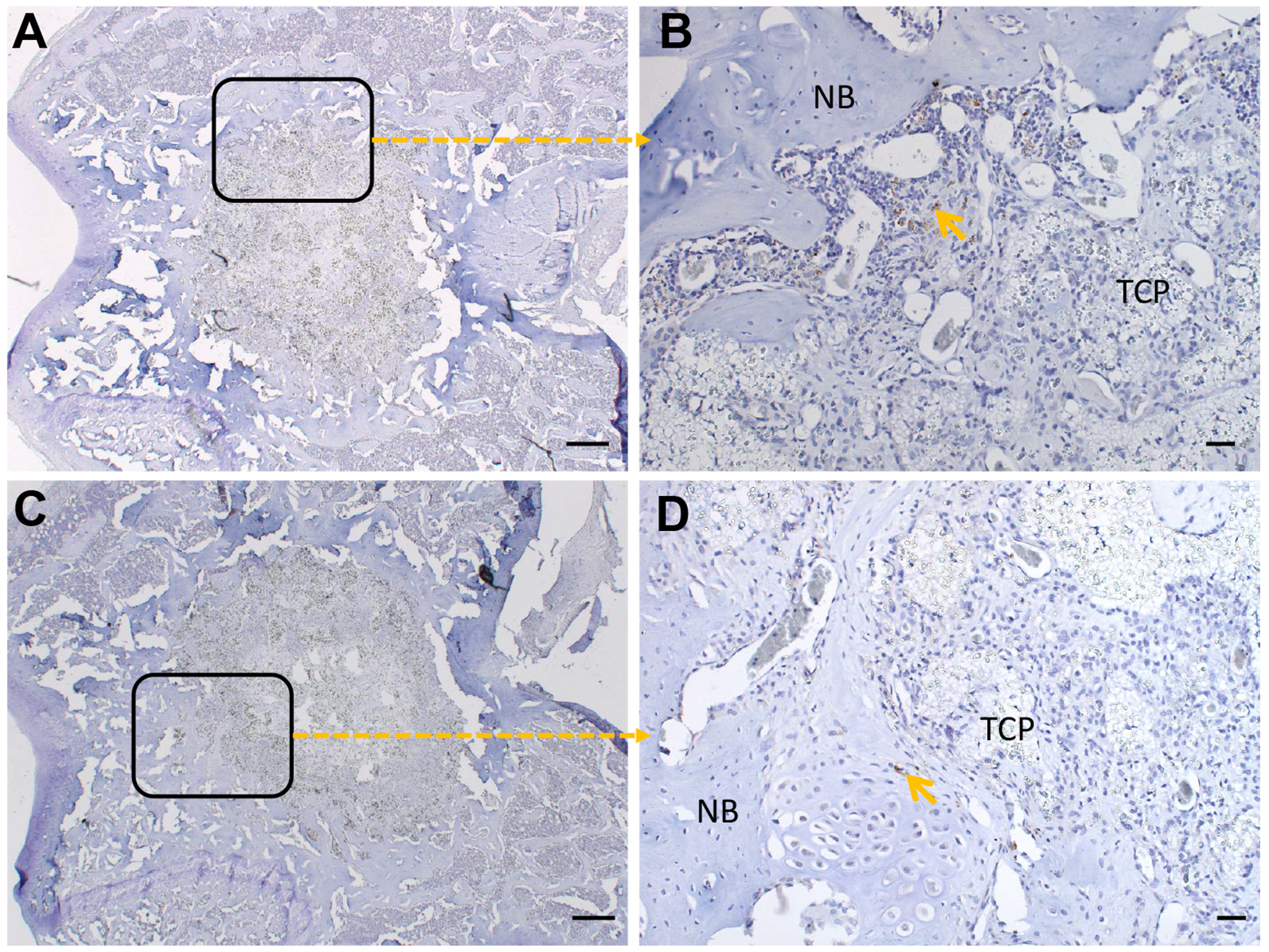

Supplement: Supplementary file 3 — Figure S3. Representative images of anti-human mitochondria staining after 4 weeks orthotopic implantation. (A) SVF constructs at low magnification; Scale bar = 500 μm. (B) SVF constructs at high magnification. NB indicates new bone and TCP indicates TCP granules. Brown arrows indicate human origin cells. Scale bar = 100 μm. (C) SVF+MO constructs at low magnification; Scale bar = 500 μm. (D) SVF+MO constructs at high magnification. NB indicates new bone and TCP indicates TCP granules. Brown arrows indicate human origin cells; scale bar = 100 μm. (TIF 5613 kb) [file 13287_2018_1026_MOESM3_ESM.tif]

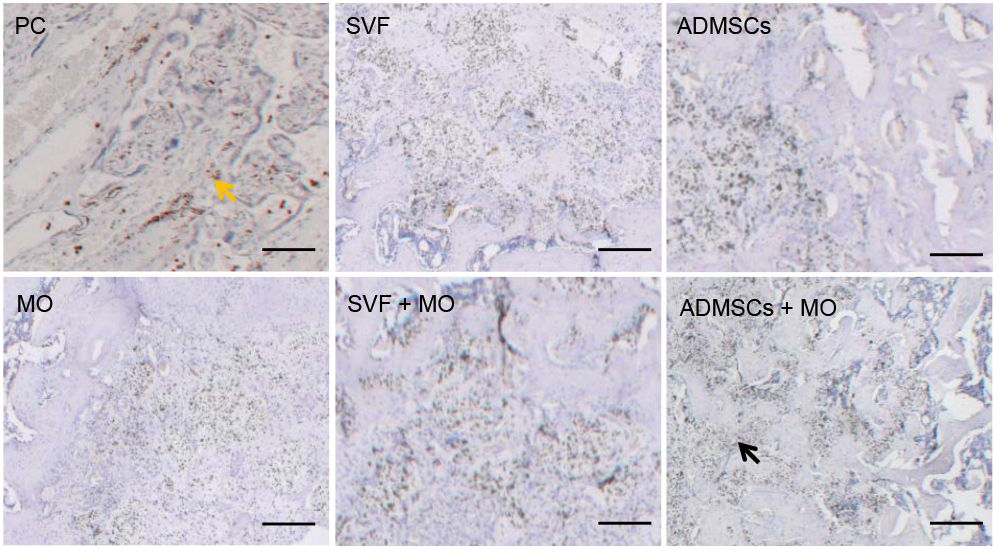

Supplement: Supplementary file 4 — Figure S4. Representative images of anti-human CD68 immunohistochemistry staining after 4 weeks orthotopic implantation. Black arrow indicates TCP granules. Yellow arrow indicates presence of human macrophages in the samples. PC indicates the positive control samples stained with anti-human CD68; Scale bar = 100 μm. (TIF 3236 kb) [file 13287_2018_1026_MOESM4_ESM.tif]

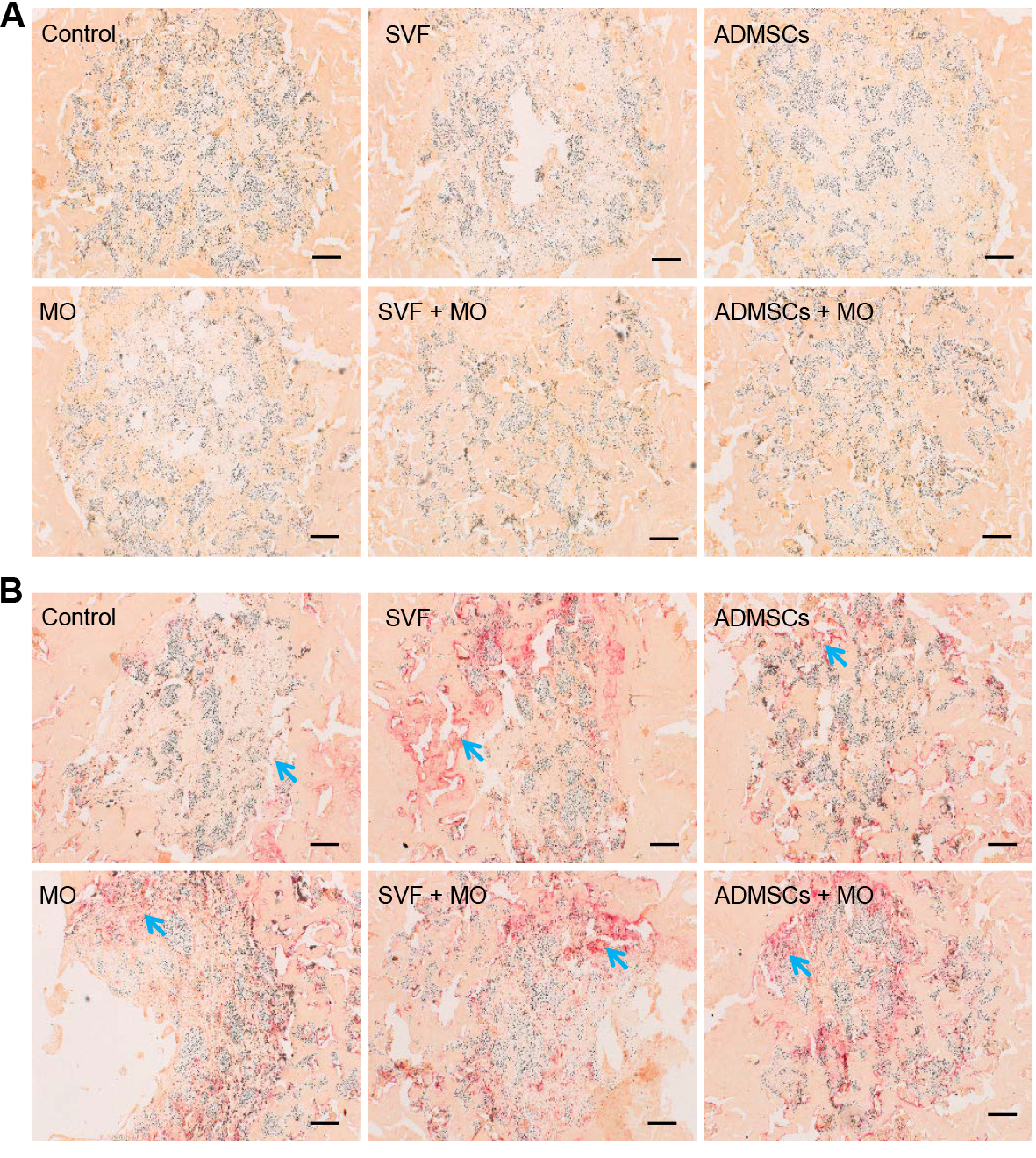

Supplement: Supplementary file 5 — Figure S5. Representative images of TRAP immunohistochemistry staining after (A) 4 and (B) 10 weeks orthotopic implantation. Blue arrows indicate TRAP-positive signals in the defect region; bar = 500 μm. (TIF 9162 kb) [file 13287_2018_1026_MOESM5_ESM.tif]
